# Meetings of Societies

**Published:** 1945

**Authors:** 


### Meetings of Societies

### Bristol Medico-Chirurgical Society

The Bristol Medico-Chirurgical Society has re-started its activities. The President (Dr. H. H. Carleton) gave his inaugural address, "Science and Nature," at the October Meeting.

The November Meeting was devoted to a discussion on the "Modern Management of Chronic Gastric and Duodenal Ulceration." The opening speech was by Mr. Norman Tanner, of London. Many members of a very large audience took part in the ensuing discussion.

The December Meeting, a Clinical one, has had to be re-arranged for 9th January, at 8 p.m., in the Out-Patient Department of the Royal Infirmary branch of the Royal Hospital.

From 1st January, 1946, Mr. Eyre Brook takes over the secretaryship from Mr. R. V. Cooke. The next Treasurer is Dr. Sutton, who takes over from Mr. Iles.

